# FFAR4 (GPR120) Signaling Is Not Required for Anti-Inflammatory and Insulin-Sensitizing Effects of Omega-3 Fatty Acids

**DOI:** 10.1155/2016/1536047

**Published:** 2016-11-24

**Authors:** Simone Isling Pærregaard, Marianne Agerholm, Annette Karen Serup, Tao Ma, Bente Kiens, Lise Madsen, Karsten Kristiansen, Benjamin Anderschou Holbech Jensen

**Affiliations:** ^1^Laboratory of Genomics and Molecular Biomedicine, Department of Biology, Faculty of Science, University of Copenhagen, Copenhagen, Denmark; ^2^The Novo Nordisk Foundation Center for Basic Metabolic Research, Section of Integrative Physiology, Faculty of Health and Medical Sciences, University of Copenhagen, Copenhagen, Denmark; ^3^Section of Molecular Physiology, Department of Nutrition, Exercise and Sports, Faculty of Science, University of Copenhagen, Copenhagen, Denmark; ^4^National Institute of Nutrition and Seafood Research, Bergen, Norway; ^5^BGI-Shenzhen, Shenzhen, China

## Abstract

Free fatty acid receptor-4 (FFAR4), also known as GPR120, has been reported to mediate the beneficial effects of omega-3 polyunsaturated fatty acids (*ω*3-PUFAs) by inducing an anti-inflammatory immune response. Thus, activation of FFAR4 has been reported to ameliorate chronic low-grade inflammation and insulin resistance accompanying obesity. However, conflicting reports on the role of FFAR4 in mediating the effects of *ω*3-PUFAs are emerging, suggesting that FFAR4 may not be the sole effector. Hence analyses of the importance of this receptor in relation to other signaling pathways and prominent effects of *ω*3-PUFAs remain to be elucidated. In the present study, we used* Ffar4* knockouts (KO) and heterozygous (HET) mice fed either low fat, low sucrose reference diet; high fat, high sucrose *ω*3-PUFA; or high fat, high sucrose *ω*6-PUFA diet for 36 weeks. We demonstrate that both KO and HET mice fed *ω*3-PUFAs were protected against obesity, hepatic triacylglycerol accumulation, and whole-body insulin resistance. Moreover, *ω*3-PUFA fed mice had increased circulating protein levels of the anti-inflammatory adipokine, adiponectin, decreased fasting insulin levels, and decreased mRNA expression of several proinflammatory molecules within visceral adipose tissue. In conclusion, we find that FFAR4 signaling is not required for the reported anti-inflammatory and insulin-sensitizing effects mediated by *ω*3-PUFAs.

## 1. Introduction

Obesity is associated with chronic low-grade inflammation causing inflammation-induced insulin resistance in various tissues [1, 2]. Overnutrition increases the need for storage of excess energy, resulting initially in adipocyte hypertrophy, later accompanied by hyperplasia, and recruitment of classically activated, proinflammatory M1 macrophages into adipose tissue [3, 4]. M1 macrophages secrete proinflammatory cytokines, recruiting additional M1 macrophages, but they also promote resident alternatively activated, anti-inflammatory M2 macrophages to differentiate towards the M1 phenotype, thereby propagating a self-amplifying vicious inflammatory cycle [4–6]. The ability of inflammation to interfere with insulin signaling was first described for tumor necrosis factor-*α* (TNF*α*) in 1993 by Hotamisligil et al. [7]. Upon binding of TNF*α*, I*κ*B kinase-*β* (IKK*β*) and c-jun N-terminal amino kinase-1 (JNK1) are activated. These serine kinases initiate proinflammatory gene transcription through activation of nuclear factor *κ*B (NF*κ*B) and activating protein-1 (AP-1), but they also have the potential to phosphorylate the insulin receptor substrates 1 and 2 (IRS1 and IRS2), inhibiting their association with the insulin receptor [1, 8, 9]. Additionally, TNF*α*-signaling upregulates expression of Suppressor of Cytokine Signaling (SOCS) protein family members, which are able to directly bind and antagonize the insulin receptor [10, 11]. Interleukin-6 (IL-6) mediated signaling similarly induces expression of proinflammatory genes and increases the expression of SOCS3 [11, 12]. Besides decreased glucose uptake into adipocytes, insulin resistance also leads to enhanced lipolysis augmenting the amount of circulating nonesterified fatty acids (NEFAs) [13]. This increases the risk for ectopic lipid accumulation in liver and muscle, further exacerbating insulin resistance, providing a link between dysfunctional adipose tissue and fatty liver [13, 14]. Yet the metabolic impact of adipose tissue inflammation varies between depots, where inflammation in visceral adipose tissue exerts a greater negative metabolic impact than inflammation in subcutaneous adipose tissue [15, 16]. Augmented secretion of cytokines, adipokines, and NEFAs, especially from visceral adipose tissue, impacts the liver, thus affecting this key metabolic organ and consequently whole-body metabolism [6, 15]. Therefore, modalities to decrease obesity-associated inflammation are of great importance. In this regard, the discovery that FFAR4 seemed to be the main receptor mediating the anti-inflammatory and insulin-sensitizing effects of *ω*3-polyunsaturated fatty acids (*ω*3-PUFAs) in adipocytes and macrophages spurred considerable interest in this receptor [17]. In recent years, the anti-inflammatory potential of FFAR4 signaling in other cell types and tissues, that is, Kupffer cells [18], colonic Caco-2 cells [19], and hypothalamus [20], has been investigated.

The anti-inflammatory effect of FFAR4 depends on the scaffold protein, *β*-arrestin 2, which upon ligand-binding of FFAR4 is recruited to the C-terminal, leading to internalization of the complex [17]. The complex is able to interfere with inflammatory signaling pathways, such as TNF*α* and toll-like receptor-4 (TLR4) mediated signaling, thereby decreasing inflammation [17]. This anti-inflammatory effect of FFAR4 was found to be responsible for the increased insulin sensitivity in a high fat diet (HFD) fed mouse model supplemented with *ω*3-PUFAs [17]. The authors ascribed the insulin-sensitizing effect of FFAR4 activation to derive from macrophages [17]. Interestingly, another group found FFAR4-deficient HFD fed mice to be more metabolically impaired, steatotic, and insulin resistant compared to their wild type (WT) counterparts independent of *ω*3-PUFA supplementation, suggesting that FFAR4* per se* has an important role in energy homeostasis [21].

Contradicting the majority of existing literature [17, 21, 22], recent evidence suggests that FFAR4 is dispensable for the beneficial effects of *ω*3-PUFAs on HFD-induced obesity [23], whereas the anti-inflammatory nature of FFAR4 remains largely unchallenged. Here we show that feeding mice a high dose of *ω*3-PUFAs protects against HFD-induced obesity, steatosis, insulin resistance, and visceral adipose tissue inflammation independent of FFAR4 status.

## 2. Materials and Methods

### 2.1. Animal Care and Use

The* Ffar4* KO mouse strain was generated by Lexicon Pharmaceuticals Inc. on a mixed background of 129SVE and C57BL/6J mice. The deleted sequence of* Ffar4* (gene accession NM_181748.2) corresponds to exon 1. Mice were bred at Taconic Laboratories and used under license (2014-15-2934-01027). All animal experiments were conducted in accordance with Danish national guidelines (Amendment #1306 of November 23, 2007) approved by the Danish Animal Inspectorate, Ministry of Justice.

Male mice were kept as mixed genotypes in cages (*n* = 3–5 per cage) under specific pathogen-free conditions at a 12 h light/dark cycle, 22-23°C, and a humidity of 30%. Mice were scaled once a week from 6 weeks of age and MR-scanned prior to the insulin tolerance test (ITT) (week 32 after diet initiation) using EchoMRI 4 in 1 (Texas, USA).

### 2.2. Diets

**Figure 1:**
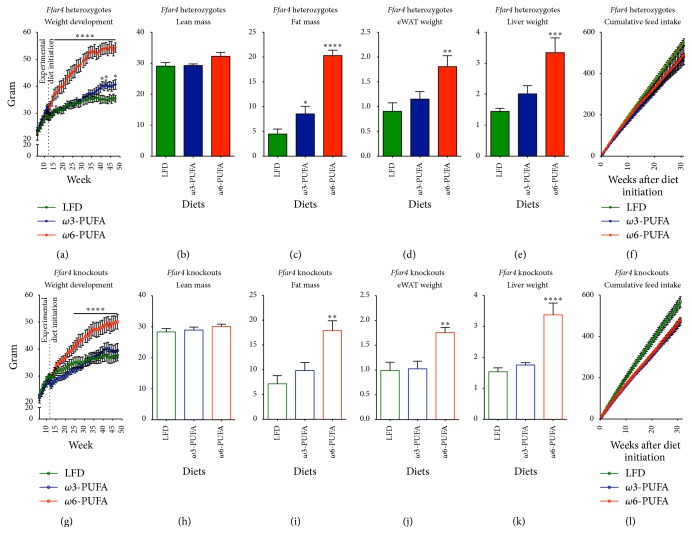
*Ffar4* HET and KO mice are protected against obesity development on a high fat *ω*3-PUFA diet. Mice were scaled weekly from 6 weeks of age; experimental diets were initiated when mice were 11 weeks old. ((a)–(f)) Heterozygotes. ((g)–(l)) Knockouts. ((a) and (g)) Weight development, *n* = 6–8. ((b) and (h)) Lean mass from MR scans 32 weeks after diet initiation (43 weeks of age), *n* = 6–8. ((c) and (i)) Fat mass from MR scans 32 weeks after diet initiation, *n* = 6–8. ((d) and (j)) eWAT weights 36 weeks after diet initiation, *n* = 6–8. ((e) and (k)) Liver weights 36 weeks after diet initiation, *n* = 6–8. ((f) and (l)) Feed intake measured two times a week in single-housed mice till 32 weeks after diet initiation, *n* = 5–7. All data are presented as means ± SEM. Both HFDs are compared to the LFD. For (a), (f), (g), and (l) data were Ln-transformed and 2-way RM ANOVA with Bonferroni correction was performed. All data shown in bar graphs were Ln-transformed and subjected to 1-way ANOVA with Bonferroni correction. Only statistical significant differences are shown. ^*∗*^ *p* < 0.05, ^*∗∗*^ *p* < 0.01, ^*∗∗∗*^ *p* < 0.001, and ^*∗∗∗∗*^ *p* < 0.0001.

Diets were obtained from Ssniff Spezialdiäten GmBH, Germany, with catalog numbers: low fat, low sucrose reference diet (S8672-E050 EF AIN93G); high fat, high sucrose fish oil diet/*ω*3-PUFA (S8672-E409 EF D12079B); and high fat, high sucrose soy oil diet/*ω*6-PUFA (S8672-E408 EF D12079B). Diets were kept at −20°C when not in use. Diet composition is shown in Table 1, with a detailed description in Table S1, in Supplementary Material available online at http://dx.doi.org/10.1155/2016/1536047. Feed intake (Figures 1(f) and 1(l)) was measured in parallel in single-housed mice fed the same diets. All other data were derived from group-housed mice. Mice were given free access to feed and water and fed fresh experimental diets twice a week from 11 weeks of age.

### 2.3. Insulin Tolerance Test (ITT)

After 33 weeks on experimental diets mice were feed-deprived for two hours prior to insulin injections (Actrapid Penfill, Novo Nordisk, Denmark). Insulin was diluted in succinylated gelatin (gelofusine, B. Braun Melsungen AG, Germany) to increase accuracy of insulin delivery. 1 U insulin per kg lean body mass was injected intraperitoneally (i.p.) after measurements of initial blood glucose concentrations. Mice were bled from the tail vein, and blood glucose was measured using the Bayer Contour Glucometer (Bayer Health Care, Germany) at indicated time points (15, 30, 45, 60, 90, and 120 minutes after injection).

### 2.4. Measurement of Insulin and Adiponectin Levels

Plasma was collected from 5-hour feed-deprived mice by tail vein bleeding and diluted two times prior to insulin measurements. For adiponectin measurements, plasma was collected from nonfasted mice by bleeding from the submandibular vein. For all plasma samples, blood was drawn in EDTA coated tubes kept on ice and centrifuged at 4°C for 10 minutes at 1000 ×g before storage at −20°C until further use. Insulin and adiponectin measurements were carried out using an electrochemiluminescence assay (Mesoscale Diagnostics, USA) following the manufacturer's instructions.

### 2.5. RNA Extraction and Quantitative RT-PCR

Liver and epididymal white adipose tissue (eWAT) were homogenized in TRIreagent (Sigma-Aldrich) using the Precellys homogenizer (Bertin Technologies). Chloroform (Sigma-Aldrich) was added for phase-separation, and RNA was precipitated by addition of isopropanol (Sigma-Aldrich) and washed with 75% ethanol (CCS Healthcare) before resuspension in autoclaved Milli-Q water. 1 *μ*g of total RNA was used for reverse transcription following manufacturer's instructions (ThermoFisher K1621). Samples were diluted in Milli-Q water and 4 *μ*L of the cDNA solution was added to a 96-well plate prior to addition of a mix consisting of 4.8 *μ*L autoclaved Milli-Q, 0.6 *μ*L forward primer (Tag Copenhagen A/S), 0.6 *μ*L reverse primer (Tag Copenhagen A/S), and 10 *μ*L SYBR Green containing ROX as reference dye (Bioline). RT-PCR was carried out on the Stratagene Mx3000P qPCR system, where samples were denatured by heating at 95°C for 5 minutes followed by 40 cycles of melting at 95°C for 15 seconds, annealing at differing temperatures as noted in Table 2 for 15 seconds, and elongation at 72°C for 20 seconds. Gene expression was normalized to that of* Tata-binding protein (Tbp)* mRNA. Primer sequences are given in Table 2.

### 2.6. Western Blot Analyses

Protein lysates were prepared from approximately 10 mg of liver tissue using standard protocols [24]. Western blot analyses were performed as previously described [24] and protein abundance was detected by immunoblotting using the following antibody: NF*κ*B p65 (Santa Cruz #sc-109). Protein concentration was measured by BCA (#23223 and #23224, Thermo Scientific, USA) according to the manufacturer's instructions. Loading consistencies were verified by Ponceau staining.

### 2.7. Thin-Layer Chromatography

Triacylglycerol (TAG) was measured by thin-layer chromatography (TLC) using 7.5 mg liver sample. Lipids were extracted in chloroform-methanol (2 : 1) using the method of Folch et al. [25] and dissolved in chloroform as previously described [26]. Lipids were separated on silica-gel coated plates using two different separate mobile phases consisting of chloroform-methanol-acetic acid-water (50 : 50 : 5 : 5) followed by petroleum ether-diethyl ether-acetic acid (120 : 25 : 1.5). Butylated hydroxytoluene (50 mg/L) was added to both of the mobile phases. The lipids were developed by a 10% copper sulfate pentahydrate and 8% phosphoric acid solution at 120°C for 15 min. Lipids were visualized on a Typhoon FLA 7000 IP fluorescent scanner and analyzed according to weight using ImageQuant TL (GE Healthcare Life Sciences, Little Chalfont, United Kingdom). TAG was identified with a specific glyceryl tripalmitate (#T5888, Sigma-Aldrich).

### 2.8. Statistics

All statistical analyses were conducted using GraphPad Prism version 6 software (GraphPad Software, San Diego, USA). Data are presented as mean ± standard error of the mean (SEM).

**Figure 2:**
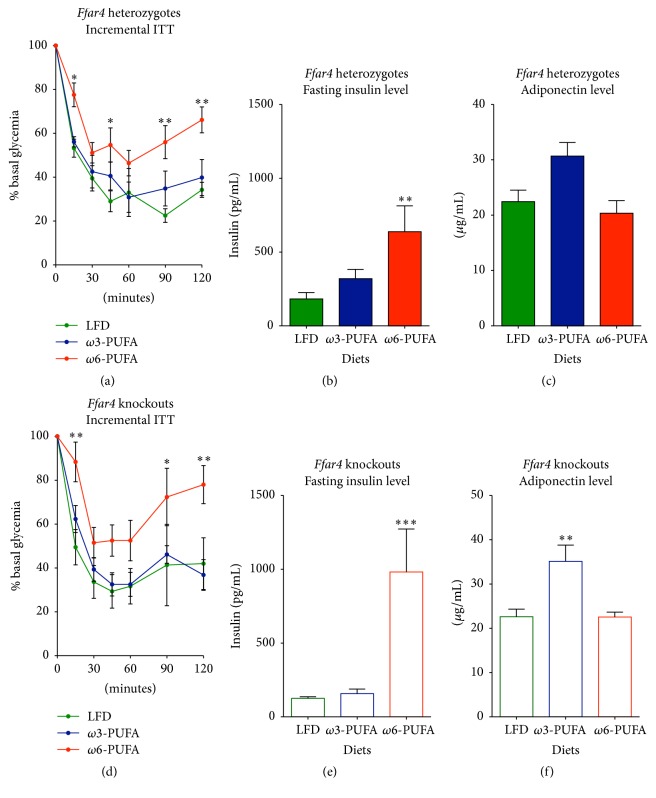
*Ffar4* HET and KO mice are protected against insulin resistance on a high fat *ω*3-PUFA diet. Insulin tolerance tests were performed at week 33 after diet initiation in 2-hour food-deprived mice. An insulin bolus of 1 U/kg lean mass was injected i.p. ((a)–(c)) Heterozygotes. ((d)–(f)) Knockouts. ((a) and (d)) ITT, *n* = 4–8; ((b) and (e)) 5-hour fasting insulin levels 32 weeks after diet initiation, *n* = 6–8. ((c) and (f)) Plasma adiponectin levels 36 weeks after diet initiation, *n* = 4–7. All data are presented as means ± SEM. Both HFDs have been compared to the LFD. For (a) and (d), 2-way RM ANOVA with Bonferroni correction was performed. All bar graph data were Ln-transformed and subjected to 1-way ANOVA with Bonferroni correction. Only statistical significant differences are shown. ^*∗*^ *p* < 0.05, ^*∗∗*^ *p* < 0.01, and ^*∗∗∗*^ *p* < 0.001.

Due to small and variable sample sizes data could not be assumed to follow a Gaussian distribution and were consequently lognormal- (Ln-) transformed prior to any test except for the ITT where data were normalized to initial blood glucose (Figures 2(a) and 2(d)). Unless otherwise noted 2-way repeated measures (RM) ANOVA with Bonferroni* post hoc* multiple comparison test was conducted for all time-dependent analyses, that is, weight development, ITT, and feed intake. For comparison between genotypes 2-way ANOVA with Bonferroni* post hoc* multiple comparison test was conducted. All other data were analyzed by 1-way ANOVA with Bonferroni* post hoc* multiple comparison test. Both HFD groups (*ω*3-PUFA and *ω*6-PUFA) were compared to the LFD group. Data were considered statistically significant with *p* < 0.05 and the different levels of significance were set to be ^*∗*^ *p* < 0.05, ^*∗∗*^ *p* < 0.01, ^*∗∗∗*^ *p* < 0.001, and ^*∗∗∗∗*^ *p* < 0.0001. Only statistically significant differences are shown on graphs.

## 3. Results

FFAR4 is currently believed to be the key receptor for the polyunsaturated long chain fatty acids, eicosapentaenoic acid (EPA), and docosahexaenoic acid (DHA) [17], mediating the beneficial effects of fish oil. Yet, reports challenging this view are emerging, suggesting that FFAR4 might not serve as the sole effector of the health beneficial effects of *ω*3-PUFAs [23], indicating that fish oil acts through multiple pathways to exert its beneficial effects on health. We examined if fish oil on a HFD background would exert a favorable metabolic effect independent of FFAR4. To address this, we acquired* Ffar4* HET and* Ffar4* KO mice from Taconic Laboratories, Denmark. Importantly, the levels of* Ffar4* mRNA were similar in WT and HET mice, showing that the expression of* Ffar4* mRNA was not gene dose dependent. No* Ffar4* mRNA was detected in KO mice (Figure S1).

We fed mice an isocaloric high fat, high sucrose diet rich in either *ω*3-PUFAs or *ω*6-PUFAs using fish oil or soy oil, respectively, as fat sources and compared the results to a low fat diet (LFD) reference group. Diet composition is shown in Table 1; a detailed fatty acid composition is given in Table S1.

### 3.1. The Antiobesogenic Effect of *ω*3-PUFAs Is Independent of FFAR4

*ω*3-PUFAs protected mice against weight gain irrespective of FFAR4 status for the first 28 weeks of HFD feeding. At this time, HET mice, but not KO mice, tended to gain more weight than their LFD fed counterparts (Figures 1(a) and 1(g)). The weight gain protection mediated by *ω*3-PUFAs is in sharp contrast to the obesogenic potential of *ω*6-PUFAs. Thus, mice, fed the latter, gained substantially more weight than LFD reference mice (Figures 1(a) and 1(g)). As evidenced by MR scans, weight gain was not confounded by increased lean mass (Figures 1(b) and 1(h)) but rather restricted to increased fat mass (Figures 1(c) and 1(i)), which was further supported by increased tissue weights of liver and eWAT (Figures 1(d)-1(e) and 1(j)-1(k)). Since FFAR4 has been shown to be implicated in fat preference [27], an effect on feed intake could be suspected. However, there was no difference in feed intake between genotypes (2-way ANOVA *p* = 0.9486) nor fat sources (Figures 1(f) and 1(l)).

### 3.2. FFAR4 Status Does Not Affect Insulin Sensitivity

The lean phenotype promoted by *ω*3-PUFAs is generally associated with increased insulin sensitivity [28]. However, in contrast to WT mice,* Ffar4* KO mice have been shown to display attenuated insulin sensitivity upon chow [17] and HFD [21] feeding. We therefore asked if* Ffar4* KO mice in the current study would develop insulin resistance in the absence of obesity. After 33 weeks on experimental diets, mice were subjected to an insulin tolerance test. Independent of genotypes (AUC, 2-way ANOVA, multiple comparison *p* > 0.9999 for all diets), *ω*3-PUFA fed mice remained equally insulin sensitive as LFD reference mice (Figures 2(a) and 2(d)). In contrast, both HET and KO mice displayed decreased insulin sensitivity (2-way ANOVA RM, main effect *p* = 0.0325 and 0.0324, resp.) and increased fasting plasma insulin concentration when fed *ω*6-PUFAs (Figures 2(a)-2(b) and 2(d)-2(e)). The protection against insulin resistance in *ω*3-PUFA fed mice was independent of FFAR4 status and correlated with increased levels of the anti-inflammatory, insulin-sensitizing adipokine, adiponectin [29] (Figures 2(c) and 2(f)), suggesting a potential mechanism for the observed protection against insulin resistance.

### 3.3. *ω*3-PUFA Fed Mice Are Less Steatotic than *ω*6-PUFA Fed Counterparts

**Figure 3:**
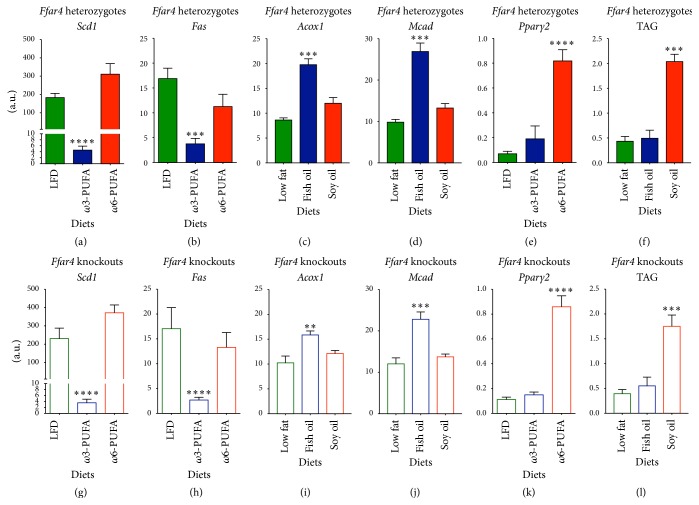
Intake of a high fat *ω*3-PUFA diet alleviates hepatic lipid accumulation in* Ffar4* HET and KO mice. Mice were euthanized in nonfasting state 36 weeks after diet initiation. Gene expression levels were analyzed by RT-qPCR and lipid levels were evaluated by thin-layer chromatography. ((a)–(f)) Heterozygotes. ((g)–(l)) Knockouts. ((a) and (g)) mRNA level of* Scd1*, *n* = 5–8. ((b) and (h)) mRNA level of* Fas*, *n* = 6–8. ((c) and (i)) mRNA level of* Acox1 n* = 6–8. ((d) and (j)) mRNA level of* Mcad*, *n* = 6–8. ((e) and (k)) mRNA level of* Pparγ2*, *n* = 6–8. ((f) and (l)) Triacylglycerol content, *n* = 6–8. Data are presented as means ± SEM. Both HFDs have been compared to the LFD. All data have been Ln-transformed and 1-way ANOVA with Bonferroni correction was performed. Only statistical significant differences are shown. ^*∗∗*^ *p* < 0.01, ^*∗∗∗*^ *p* < 0.001, and ^*∗∗∗∗*^ *p* < 0.0001.

**Figure 4:**
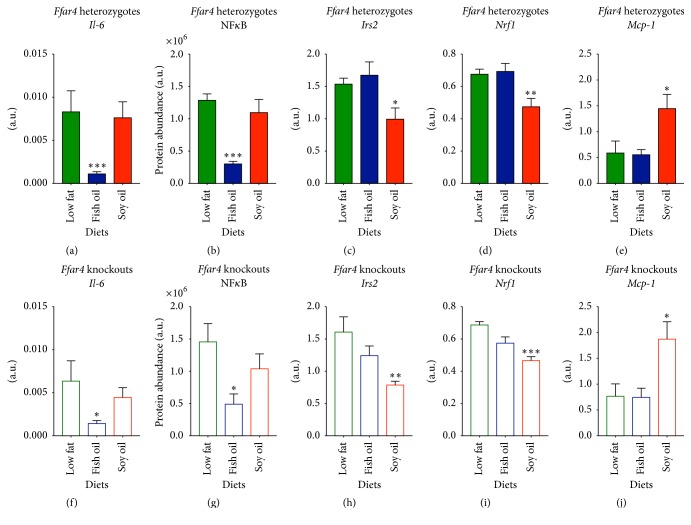
Intake of a high fat *ω*3-PUFA diet alleviates hepatic steatosis in* Ffar4* HET and KO mice. Mice were euthanized in nonfasting state 36 weeks after diet initiation. Gene expression levels were analyzed by RT-qPCR and protein levels were evaluated by western blot analysis. ((a)–(e)) Heterozygotes. ((f)–(j)) Knockouts. ((a) and (f)) mRNA level of* Il*-*6*,  *n* = 5–7. ((b) and (g)) Protein level of NF*κ*B, *n* = 6–8. ((c) and (h)) mRNA level of* Irs2*, *n* = 6–8. ((d) and (i)) mRNA level of* Nrf1*, *n* = 6–8. ((e) and (j)) mRNA level of* Mcp*-*1*, *n* = 6–8. Data are presented as means ± SEM. Both HFDs have been compared to the LFD. All data have been Ln-transformed and 1-way ANOVA with Bonferroni correction was performed. Only statistical significant differences are shown. ^*∗*^ *p* < 0.05, ^*∗∗*^ *p* < 0.01, and ^*∗∗∗*^ *p* < 0.001.

To disentangle the insulin-sensitizing, FFAR4 independent effects of *ω*3-PUFAs, we investigated hepatic expressions of lipogenic enzymes. Notably, expression of* Stearoyl coenzyme A desaturase-1 (Scd1)*, encoding an enzyme required for diet-induced hepatic insulin resistance [30], was substantially downregulated in *ω*3-PUFA fed mice compared to LFD reference mice (Figures 3(a) and 3(g)). Similarly, expression of* fatty acid synthase (Fas)* was significantly decreased in *ω*3-PUFA fed mice (Figures 3(b) and 3(h)) with a concomitant increased expression of genes involved in fatty acid oxidation,* acyl-CoA oxidase 1 (Acox1)* and* medium-chain acyl-CoA dehydrogenase (Mcad)* (Figures 3(c)-3(d), 3(i)-3(j)). This suggests that hepatic* de novo* lipogenesis was diminished by *ω*3-PUFA feeding, regardless of FFAR4 status. Moreover, expression of the adipogenic marker,* peroxisome proliferator-activated receptor-γ*2* (Pparγ2)*, was significantly increased in *ω*6-PUFA fed mice compared to LFD reference mice (Figures 3(e) and 3(k)), possibly reflecting increased fat storage capacity [31]. In keeping with this notion, we observed increased levels of hepatic TAG accumulation (Figures 3(f) and 3(l)). In line with the observed decreased* de novo* lipogenesis, improved hepatic insulin resistance, and reduced TAG accumulation in *ω*3 compared with *ω*6-PUFA fed mice, hepatic* Il-6* expression as well as the protein level of the proinflammatory nuclear factor, NF*κ*B, was diminished in *ω*3-PUFA fed mice. Further, expressions of* insulin receptor substrate 2 (Irs2)* and* NF-E2-related factor 1 (Nrf1)* were lower in *ω*6-PUFA fed mice concurrent with augmented expression of* macrophage chemoattractant protein-1 (MCP-1)* (Figures 4(a)–4(j)). Again, no differences between genotypes on either diet were observed suggesting an independency of FFAR4 in both the protection and progression of hepatic steatosis.

### 3.4. *ω*3-PUFA Fed Mice Show Signs of Decreased Inflammation in the Visceral Adipose Tissue

**Figure 5:**
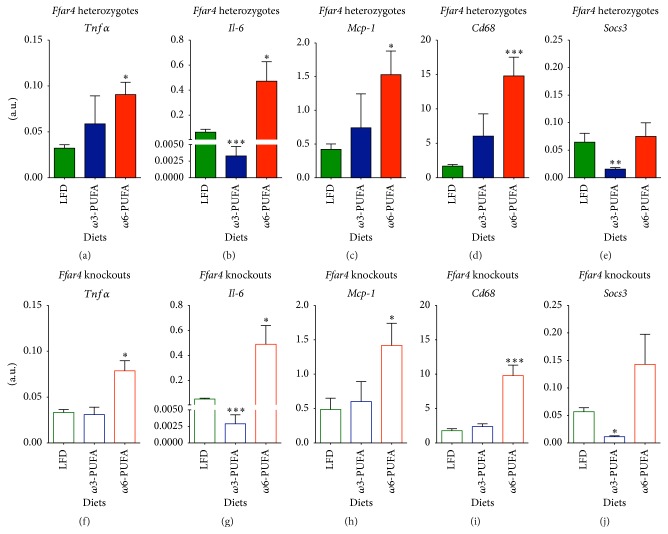
Inflammatory status of eWAT is indistinguishable between* Ffar4* HET and KO mice. Mice were euthanized in nonfasting state 36 weeks after diet initiation. Gene expression levels were analyzed by RT-qPCR. ((a)–(e)) Heterozygotes. ((f)–(j)) Knockouts. ((a) and (f)) mRNA level of* Tnfα*, *n* = 4–8. ((b) and (g)) mRNA level of* Il-6*, *n* = 4–8. ((c) and (h)) mRNA level of* Mcp-1*, *n* = 4–8. ((d) and (i)) mRNA level of* Cd68*, *n* = 4–8. ((e) and (j)) mRNA level of* Socs3*, *n* = 4–8. Data are presented as means ± SEM. Both HFDs have been compared to the LFD. All data have been Ln-transformed and subjected to 1-way ANOVA with Bonferroni correction. ^*∗*^ *p* < 0.05, ^*∗∗*^ *p* < 0.01, and ^*∗∗∗*^ *p* < 0.001.

Immunometabolism, hallmarked by tissue cross talk, has attracted considerable attention over the past decade. Adipose tissue harbors multiple immune cells [32] while adipocytes themselves have substantial immunomodulatory capacity [6]. FFAR4 has been shown to promote its positive insulin-sensitizing effect by inhibiting macrophage-mediated inflammation in adipose tissue [17], and since visceral adipose tissue exerts a larger impact on whole-body metabolism than subcutaneous fat [15, 16] we focused our immunological analyses on the former. To investigate whether inflammation and macrophage recruitment were altered between diets and genotypes, we measured gene expression levels of* Tnfα*,* Il-6*,* Mcp-1*,* Cd68,* and* Socs3* in eWAT of* Ffar4* HET and KO mice (Figure 5). The proinflammatory cytokine,* Tnfα*, was significantly increased in *ω*6-PUFA fed mice compared to LFD reference mice (Figures 5(a) and 5(f)). Interestingly, the expression levels of* Il-6* were substantially reduced in *ω*3-PUFA fed mice of both genotypes compared to their LFD fed counterparts (Figures 5(b) and 5(g)), suggesting a FFAR4 independent mechanism for at least some anti-inflammatory effects of *ω*3-PUFAs. Obesity-associated low-grade inflammation is characterized by increased macrophage accumulation in adipose tissue [5] and increased expression of* Mcp-1* is hypothesized to account for this increase [4]. While others have found that *ω*3-PUFA supplementation suppressed the expression of* Mcp-1* in adipose tissue of* Ffar4* WT mice but not* Ffar4* KO mice [17], we found no differences in* Mcp-1* expression between genotypes (2-way ANOVA *p* = 0.8274). Further, the expression of* Mcp-1* was indistinguishable between *ω*3-PUFA fed mice and LFD reference mice, whereas *ω*6-PUFA fed mice had an augmented* Mcp-1* expression (Figures 5(c) and 5(h)) concomitant with increased expression of the global macrophage marker,* Cd68* (Figures 5(d) and 5(i)), indicating an augmented infiltration of macrophages in adipose tissue of *ω*6-PUFA fed mice. Lastly, as a marker of general tissue inflammation, we analyzed the expression levels of* Socs3*. Strikingly, the finding mirrored the expression levels of* Il-6* with a selective reduction of* Socs3* expression in *ω*3-PUFA fed mice (Figures 5(e) and 5(j)) independent of FFAR4 status, further supporting the notion of diminished inflammation and implying that FFAR4-independent pathways may confer beneficial effects of *ω*3-PUFAs.

## 4. Discussion

The potential of fish oil to protect against cardiovascular diseases is well-established [33]. In recent years, however, there has been an increasing interest in the ability of fish oil to relieve other lifestyle diseases such as obesity and type 2 diabetes. Although human studies are inconclusive, the antiobesogenic potential of fish oil in rodents is well-documented [34, 35]. Still, the molecular mechanisms by which *ω*3-PUFAs mediate their actions are intensely debated [17, 28, 36–41]. Several mechanisms have been proposed to explain the beneficial effects of *ω*3-PUFA supplementation, comprising increased fatty acid oxidation [28, 38] and anti-inflammatory actions [17, 40, 42], alleviating insulin resistance and metabolic syndrome [43]. On this note, FFAR4 was recently reported to be responsible for the anti-inflammatory and insulin-sensitizing effects of *ω*3-PUFAs [17]. This finding led us to investigate whether fish oil on a background of high fat, high sucrose diet would improve metabolic parameters in* Ffar4* KO mice to the same extent as observed in WT mice. We compared the results to LFD reference group and further employed an obesogenic HFD control where the fat source was based on soy oil, rich in *ω*6-PUFAs. Importantly, the *ω*6-PUFA in soy oil is linoleic acid (LA), which parallels *ω*3-PUFAs in the ability to agonize FFAR4 [44].

We found that the decreased liver weights of *ω*3-PUFA fed mice were paralleled by decreased expressions of genes encoding the lipogenic enzymes,* Fas* and* Scd1*, possibly due to suppression of processing or activity of SREBP1c [37, 45], and an increased expression of genes involved in fatty acid oxidation,* Acox1* and* Mcad*. Moreover, expression of* Nrf1*, a transcription factor protecting against hepatic steatosis [46], was selectively decreased in *ω*6-PUFA fed mice, while* Pparγ2* was increased; the latter is possibly reflecting a requirement for increased fat storage [31], which was further supported by increased TAG accumulation in the livers of these mice. The protection against weight gain and liver lipogenesis and adipogenesis in *ω*3-PUFA fed mice was associated with improved insulin sensitivity as determined by an ITT. The improved insulin sensitivity was further supported by lowered fasting plasma insulin and augmented plasma adiponectin. Importantly, both the beneficial effects of *ω*3-PUFA feeding and the detrimental effects of *ω*6-PUFA feeding were independent of genotype. These findings reflect a recent study focusing on energy metabolism and energy expenditure which questioned the necessity of FFAR4 signaling in fish oil-mediated health benefits [23]. Yet, the anti-inflammatory action of FFAR4-mediated signaling, as reported by Oh et al. [17], has so far remained unchallenged. Accordingly, we investigated the inflammatory status of liver and visceral fat (eWAT), where a potential anti-inflammatory effect may exert a major impact on whole-body metabolism. Surprisingly, we found decreased expression of inflammatory genes and proteins in *ω*3-PUFA fed mice irrespective of genotypes suggesting that FFAR4 is dispensable for the immunometabolic effects of *ω*3-PUFAs. This is in sharp contrast to the findings of Oh et al., who found decreased expression of* Il-6* and* Mcp-1* solely in WT mice, but not in* Ffar4* KO mice, fed a *ω*3-PUFA enriched HFD [17]. The different outcomes of the studies performed by Oh et al. [17] and those reported here may relate to subtle differences in the experimental setup. Both studies were performed on mice of mixed 129SVE and C57BL/6J backgrounds, but it is unclear to what extent the mice of the Oh et al. study had been backcrossed to the C57BL/6J background [17]. This could have a vast impact on the immunological outcomes of these studies, since C57BL/6J and 129SVE mice have different inflammatory responses [47]. Furthermore, our HET and KO mice were cocaged throughout the study. It has been shown that the microbiota in some instances might exert a larger impact on phenotype compared to genotype [48]. Accordingly, it is indeed possible that the effect of cocaging, hence exposing* Ffar4* KO mice to microbiota from HET mice, had masked the effect of* Ffar4* ablation.

Collectively, our findings demonstrate that *ω*3-PUFAs may exert positive effects independently of FFAR4 or at least that the effect of FFAR4 is minor in the setting of a high fat fish oil-based diet. This is not to question the well-described anti-inflammatory and insulin-enhancing potential of FFAR4 [17, 22] but merely an indication of the fact that there might be a certain level of redundancy of the said receptor and that *ω*3-PUFAs may have multiple undiscovered receptors through which they exert their beneficial actions. Indeed, *ω*3-PUFAs impact a myriad of metabolic processes, and the extent to which FFAR4 signaling is involved remains to be elucidated. Besides increasing adiponectin secretion [36], reported here to be independent of FFAR4, EPA and DHA serve as precursors for bioactive lipid mediators such as eicosanoids/docosanoids [49], resolvins [40], maresins [50], and protectins [51]. These compounds have anti-inflammatory effects and may potentially curb HFD-mediated low-grade inflammation, thereby relieving insulin resistance. Moreover, EPA and DHA-derived prostanoids are considered less proinflammatory than those derived from arachidonic acid (AA) [52]. Competition between *ω*3-PUFAs and AA for incorporation into phospholipids furthermore reduces substrate availability for synthesis of a number of oxylipins [53] as well as the two major endocannabinoids [54, 55]. The importance of such lipid mediators in relation to FFAR4-dependent signaling remains to be established. It has been shown that the beneficial effects on hepatic steatosis and adipose tissue insulin sensitivity by supplementing ob/ob mice with *ω*3-PUFAs were due to increased levels of protectin D1 and resolvin D1 [40]. These mediators have not been investigated in the present study, and future studies are needed to explore to what extent such lipid mediators contribute to the beneficial effects associated with fish oil intake. Additional candidates involved in *ω*3-PUFA signaling may comprise other G-protein coupled receptors, for example, FFAR1 and GPR119. Indeed, FFAR1 has been shown to partly mediate the anti-inflammatory effects of DHA by inhibition of inflammasome activation, where only* Ffar4*/*Ffar1* double KO abrogated this effect [56]. Hence, the beneficial effects of high dose *ω*3-PUFAs may also in part be mediated by FFAR1. Moreover, DHA, EPA, and their derivatives can activate PPARs, which collectively have been found to be able to inhibit inflammation through repression of NF*κ*B activation [57]. Furthermore, adiponectin secretion has been shown to be PPAR*γ*-dependent [36], while PPAR*α* activation leads to increased fatty acid oxidation [57], thus establishing PPARs as important mediators and possible effectors of the *ω*3-PUFA-mediated FFAR4 independent effects described in the present study.

In conclusion, our data provide evidence for alternative routes, not dependent on FFAR4, involved in mediating the beneficial effect of *ω*3-PUFAs, and emphasize the importance of *ω*3-PUFAs in relation to adequate immune regulation in curtailing the metabolic syndrome.

## Supplementary Material

The Supplementary Material indcludes figure S1 depicting gene expression levels of ffar4 in WT, HET and KO mice, corrorborating that gene expression levels of ffar4 WT and HET mice are similar. Furthermore a detailed description of the macro- and micronutrient composition of the different diets are shown in Table S1.

## Figures and Tables

**Table 1:** Diet composition of the three different diets. A detailed description can be found in supplementary Table S1.

	Diet composition		
	Low fat	Fish oil	Soy oil
kcal/g	3.82	4.54	4.54
Protein (kcal%)	19	15	15
Fat (kcal%)	17	42	42
*ω3-PUFA* *(% of total fat)*	*0.82* *(4.82)*	*14.9* *(35.48)*	*2.5* *(5.95)*
*ω6-PUFA* *(% of total fat)*	*7.63* *(44.88)*	*1.0* *(2.38)*	*22.75* *(54.17)*
*ω6 : ω3 ratio*	*9.31 : 1*	*0.07 : 1*	*9.10 : 1*
Carbohydrates (kcal%)	64	43	43
*Sucrose* *(% of total carbohydrate)*	*13.5* *(21,10)*	*29.5* *(60.60)*	*29.5* *(60.60)*

**Table 2:** Primer sequences and annealing temperatures. Primer sequences and annealing temperature are depicted for relevant primer pairs.

Primers	Primer (forward, reverse)	Annealing temp.
Acox1	5′GGGTCATGGAACTCATCTTCGA	58°C
	5′GAATGAACTCTTGGGTCTTGGG	

Cd68	5′CTTCCCACAGGCAGCACAG	61°C
	5′AATGATGAGAGGCAGCAAGAGG	

Fas	5′ATTGGTGGTGTGGACATGGTC	61°C
	5′CCCAGCCTTCCATCTCCTG	

Il-6	5′CTCTGCAAGAGACTTCCATCCAGT	60°C
	5′GAAGTGGTATAGACAGGTCTGTTGG	

Irs2	5′TCTGCCAGCACCTATGCAA	60°C
	5′GCTTCACTCTTTCACGACTGTG	

Mcad	5′AGTATGCCCTGGATAGGAAGACAT	60°C
	5′CTTGGTGCTCCACTAGCAGCT	

Mcp-1	5′GTGTTGGCTCAGCCAGATGC	62°C
	5′GCTTGGTGACAAAAACTACAGC	

Nrf-1	5′CAGCACCTTTGGAGAATGTG	55°C
	5′CCTGGGTCATTTTGTCCACA	

Ppar*γ*2	5′ACAGCAAATCTCTGTTTTATGC	60°C
	5′TGCTGGAGAAATCAACTGTGG	

Scd1	5′ACACCTGCCTCTTCGGGATT	61°C
	5′TGATGCCCAGAGCGCTG	

Socs3	5′GCCTTTCAGTGCAGAGTAGTG	63°C
	5′AAGAGCAGGCGAGTGTAGAG	

Tbp	5′ACCCTTCACCAATGACTCCTATG	60°C
	5′ATGATGACTGCAGCAAATCGC	

Tnf*α*	5′CCCTCACACTCAGATCATCTTCT	63°C
	5′GCTACGACGTGGGCTACAG	
