# Supplementary material for: FFAR4 (GPR120) Signaling Is Not Required for Anti-Inflammatory and Insulin-Sensitizing Effects of Omega-3 Fatty Acids
Source: Mediators Inflamm. 2016 Nov 24;2016:1536047. doi: 10.1155/2016/1536047 (PMC5143742; doi:10.1155/2016/1536047)
Supplement: Supplementary file 1 — The Supplementary Material indcludes figure S1 depicting gene expression levels of ffar4 in WT, HET and KO mice, corrorborating that gene expression levels of ffar4 WT and HET mice are similar. Furthermore a detailed description of the macro- and micronutrient composition of the different diets are shown in Table S1. [file 1536047.f1.pdf]

Supplementary figure and table legends

Supplementary figure 1 *Ffar4* WT and HET mice exhibit similar *Ffar4* mRNA levels.

Ffar4 mRNA level measured by RT-qPCR, n= 18-20..

Data are presented as means ± SEM. All genotypes have been compared to the KO. 1-way ANOVA with Bonferroni correction. ****$p<0.0001$.

Supplementary Table 1 Detailed diet compositions

Unless otherwise noted, percentages describe the weight percentage.

Supplementary Figures and Tables

Supplementary Figure 1

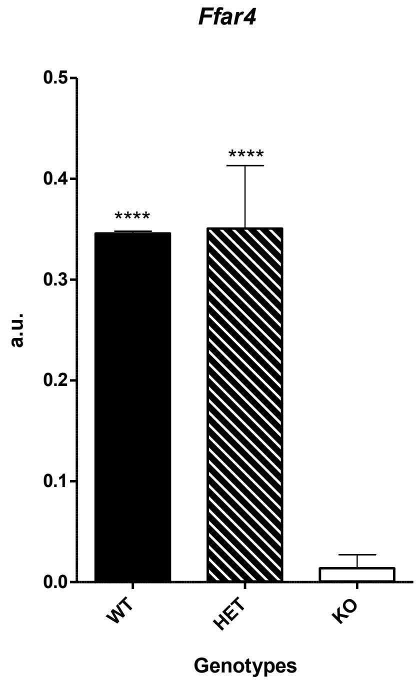

Supplementary Table 1

Product No. *(customized)*		S8672-E0500 AIN mod. LF Control	S8672-E408 AIN, SBO Oil High Sucrose	S8672-E409 AIN, Fish Oil High Sucrose
	**Weight %**			
Casein	%	20,0000	19,5000	19,5000
Corn starch	%	52,0000	5,0000	5,0000
Maltodextrin	%	——	10,0000	10,0000
Sucrose	%	9,7770	31,6270	31,6270
Cellulose powder	%	5,0000	5,0000	5,0000
L-Cystine	%	0,3000	0,3000	0,3000
DL-Methionine	%	0,1600	0,1600	0,1600
Vitamin premix	%	1,0000	1,0000	1,0000
Mineral premix	%	3,5000	6,0000	6,0000
Choline chloride	%	0,2500	0,2500	0,2500
Vitamin K3	%	0,0005	0,0005	0,0005
Vitamin B12	%	0,0025	0,0025	0,0025
Butylated hydroxytoluene	%	0,0100	0,0100	0,0100
Cholesterol	%	——	0,1500	0,1500
Na CMC	%	1,0000	——	——
Soybean oil	%	7,0000	21,0000	——
Fish oil	%	——	——	21,0000
Σ		100,000	100,000	100,000
Crude protein	%	17,8	17,3	17,3
Crude fat	%	7,1	21,1	21,1
Crude fibre	%	5,0	5,0	5,0
Crude ash	%	3,4	5,4	5,4
Starch	%	50,0	4,8	4,8
Sugar		10,9	32,5	32,5
	**Energy %**			
ME (Atwater) MJ/kg		16,0	19,0	19,0
Protein	%	19	15	15
Fat	%	17	42	42
CHO	%	64	43	43
Fatty acids in the diet	**Weight %**			
C14:0	%	0,02	0,02	1,59
C15:0	%	——	——	0,12
C16:0	%	0,79	2,44	3,90
C17:0	%	——	——	0,13
C18:0	%	0,25	0,75	0,82
C16:1	%	0,01	0,02	1,77
C18:1	%	1,78	5,27	2,40
C18:2	%	3,71	11,06	0,30
C18:3	%	0,40	1,22	0,16
C18:4	%	0,01	0,02	0,64
C20:0	%	0,03	0,08	0,12
C20:1	%	0,02	0,05	0,44
C20:4	%	——	——	0,19
C20:5	%	——	——	3,68
C22:1	%	——	——	0,09
C22:5	%	——	——	0,41
C22:6	%	——	——	2,54
